# Public Hospitals and Their Maintenance
*The first part of this article was published in The Hospital of March 25, p. 751.


**Published:** 1911-04-29

**Authors:** R. Chambers Norman

**Affiliations:** Secretary-Superintendent of the Alfred Hospital, Melbourne.


### SPECIAL INSTITUTIONAL ARTICLE.

### II.—PUBLIC HOSPITALS AND THEIR MAINTENANCE.*

By R. CHAMBERS NORMAN, Secretary-Superintendent of the Alfred Hospital, Melbourne.

### II.—HOSPITAL MAINTENANCE.

During the past quarter of a century not only has there been an increase in the number of our metropolitan hospitals but also in the accommodation provided at the previously existing ones. I fear there has been a tendency in the past to extend hospital accommodation, while financial considerations for the efficient maintenance of this increased accommodation has not received the attention it should have done. Now, I hold the same opinion as Sir H. Burdett, when he says—" No population is justified in incurring a large expenditure upon hospital buildings unless it is prepared to maintain those buildings in efficiency, and fully occupied."

#### Government Sanction.

It is one thing to start an institution on a flood of enthusiasm and with a " grand flourish of trumpets," but quite another to maintain that charity year in and year out at its best; and it does not require one to be a Solomon to know which is the easier. The Government, if not the general public, has " tumbled to " the situation, for some time ago an edict was issued that no State subsidised institution shall expend over £100 on building without first having obtained the Government's approval.

Well, now, let us look into hospital expenditure first, though the usual practice in balance-sheet reading is to take receipts first. There is an old saw—" a pound saved is a pound earned." If therefore I can indicate directions in which economies might be effected without prejudice to the patients it will be a direct gain to the cause of charity.

I think you will hold with me that nothing that has to be conserved should be wasted. It annoys me to see a water-tap that has not been turned off. while annoyance becomes anger when I find gas burning without the slightest occasion for its use.

#### Some Cheaper Substitutes.

In conversation with one and other members of my hon. medical staff, the substitution of cocoa for the ancient custom which still prevails of beef tea all round at 11 a.m., has more than once been discussed. If this change were carried out in the case of every patient at the Alfred Hospital for one year (168 beds) it would effect a net saving of over £200, and the patients would like cocoa just as well as beef-tea, and it would do them quite as much good. But remember, I cannot speak with authority as regards the latter, for I am not a doctor.

Then if the doctors at our hospitals could see their way to use a cheap thing like oakum for padding the splints used for simple fractures, instead of cotton wool, considerable saving might be effected. But after all, it is not so much the use of cheap lines that I would urge as the more economical use of expensive things. Colman, the great mustard maker, is said to have told someone that he had made his fortune more out of the mustard wasted than what was actually consumed.

Regarding the use of stimulants I am thankful to say that the necessity for protest has passed. In the good old times we used over 3000 ounces of brandy, that is about 20 gallons, for 237 patients in one month, while my last month's returns were 915 ounces for 308 patients. Figures like these seem to indicate either that formerly too much brandy was prescribed or that now too little is given. I incline to the former opinion! Going back to the year 1892 I remember discovering on September 30 that a patient had been on 8 ounces of brandy a day since soon after his admission on July 18 previous, which was over two bottles of brandy a week for ten weeks! It came about in this way. After a severe operation the surgeon told the R.M.O. to " pitch the stimulants into the patient," meaning of course only for such time as the man's vitality was at a low ebb. But the resident kept on " pitching the stimulants in " until my attention was called to the case by the clerk when writing up the " Stimulant Book."

#### Growth of Expenditure.

Compared with former years, hospital expenditure on surgical instruments, appliances, and dressings, etc., is heavy. To give an example—modern surgery, or, it might be more correct to say, many surgeons, deem the use of operating gloves essential, very often as many pairs of gloves are " done for " as there are operations performed, and these gloves cost up to 4s. or 5s. a pair. I am hoping, however, that this expensive practice has not come to stay. Doctors, like everyone else, have their fads, and operating with gloves, on all occasions, is one.

Piling on " extras " in the shape of food and drink soon runs away with the money. What do you think of the following extract from a patient's diet card, in addition to the usual full meal diet: " 6 eggs daily, also custard, 1 dozen oysters, 2 pints of milk, 2 bottles of lemonade, 1 pint bottle of ale, and 2 ounces of brandy." And yet in spite of this generous quantity of meat and drink the patient actually died.

Can you be surprised that professional " deadbeats " and " sundowners " liked to winter in a hospital?

My long experience has convinced me that more or less waste and extravagance are inseparable from hospitals, more particularly large ones; but if all hospital employés, from the highest to the lowest, would only be as economical in the use of fuel, light, food, and other materials as they would be if they had to pay for these things out of their own pockets, what a wonderful saving of money could be effected! Alas! no power in heaven or on earth appears able to make some people careful of other people's property.

---

* The first part of this article was published in The Hospital of March 25, p. 751.

And now we come to that vexed question of "cost of administration" over which there was such an outcry four years ago. First of all let us understand what we mean by the term. In the Burdett system of hospital book-keeping, which by direction of the Government all hospitals in Victoria have adopted, "administration" includes the following: "Official and clerical salaries, collectors' salaries and commission, official printing, stationery, postages and advertising, law charges, interest on bank overdraft, fidelity guarantee premiums, auditors' fees, and travelling expenses." Now the moot point is, what proportion should the aggregate of these charges bear to the total amount expended on maintenance?

The Victorian Government first investigated and published this cost of administration in the year 1901-02, when the then inspector of charities (Mr. Short) in his annual report quoted the following remarks on the subject by Sir Henry Burdett: "For metropolitan general hospitals the cost of administration should not exceed as a maximum 15 per cent. of the cost of maintenance, while in the provinces experience proves that from 10 to 12 per cent. is the highest outlay. These calculations relate to hospitals containing a hundred beds and upwards."

Now four years later when all the fuss arose, the cost of administration at the Alfred Hospital was 9 per cent., as shown in the Inspector of Charities' report for that year. And yet the late Sir T. Bent stated in reply to a deputation from that hospital: "The amount of money spent on administration is alarming. I find it costs 76 per cent. of the total sum contributed to work our hospitals." As a matter of fact, what the ex-Premier really found was "a mare's nest," as articles in the Press quickly demonstrated.

### How the Money Might be Raised.

How are the necessary funds to be raised that will maintain our medical charities in a state of absolute efficiency without pressing unduly on any one section of the community? In considering this question, I assume that you will agree with me that hospitals should not be placed in the same category as benevolent asylums, homes for the aged and infirm, or convalescent and similar institutions, inasmuch as large metropolitan hospitals cost a great deal more to maintain, and are schools of instruction, affording the most valuable experience obtainable, alike for medical men, students, and nurses.

The benefits they confer are not therefore restricted to the relief of pain or the cure of disease, though these latter are of course their *raison d'être*, and naturally appeal most strongly to our sympathies. Regarded in the broader sense, a well constructed and efficient hospital is a desideratum in any community, as it benefits directly or indirectly every member thereof. Hence its maintenance should be recognised as a public obligation, and not depend on the willingness and ability to contribute of a comparatively small number of benevolent people, but rather on some system of taxation that will reach every pocket, even if the heart remain untouched.

We hear a lot about the tendency that such a system would have "to dry up the stream of charity," but there surely would remain, after our medical institutions had become independent of that stream, plenty of dry places for its fertilising and refreshing influence to make itself felt.

With the advance of science, discoveries are constantly being made in curative medicines and surgery (take for example the use of radium), but alas! the cost of some of these desiderata puts them beyond the means of all save a few to procure and utilise, and thus the best results cannot be obtained.

Now, though I readily admit that you cannot make people either virtuous, charitable, or just by Acts of Parliament, still one can go a considerable distance in the last direction if the class for whose benefit our public hospitals have been erected and equipped were required by law to help to maintain them in some such manner as follows:—

1. That all wage-earners shall contribute to a medical charities fund every week or month, according as they are paid, on the following scale, namely: Those receiving less than 10s. a week to be exempt; 10s. and under 20s. a halfpenny; 20s. and under 30s. a penny; between 30s. and 40s. a week, 1½d.; 40s. to 50s., 2d.; 50s. and under 60s., 3d. Where wages are paid monthly, four times these amounts shall be deducted from each person's earnings, so that practically 1s. a month would be the highest tax demanded, and that only from persons in receipt of £10 to £12 a month.

2. That these contributions to the medical charities fund be deducted each pay-day from every employé's wages by the employer or his agent, and that a return of the amounts thus received be filled in on statutory printed forms to be supplied by the Government free of cost. I need not trouble you now with the details for carrying out this scheme, though I furnished some in the letter from which I quote.

3. That in order to bring under the medical charities tax those engaged in domestic service, all labour agents and registry office keepers shall procure what might be termed "Charity Tax Stamps," having the respective values of 3d., 6d. and 1s., and in every case of an engagement an agreement to accept service shall be signed by the employé, and have a stamp affixed thereto for which the employé must pay on the following scale: Servants engaged at less than 12s. a week, 3d.; 12s. to 15s., 6d.; over 15s., 1s. Any agent failing to affix such stamp and any employé refusing to pay the cost be deemed guilty of a misdemeanour.

We come now to those whose incomes amount to £150 a year and upwards. These should be reached by an income-tax (specially ear-marked) of one penny in the pound without any exemption, and no matter from what source obtained; so that the man or woman whose income was £150 would pay a medical charities tax of 12s. 6d. a year; if £500 a year, £2 1s. 8d.; if £1,000, £4 3s. 4d., and so on up to the highest paid official, and the biggest fee-earning doctor or lawyer in the State.

